# POCUS-guided fluid removal on haemodialysis using LVOT VTI in a patient with complex hemodynamics, case report

**DOI:** 10.1186/s12882-026-04870-9

**Published:** 2026-03-12

**Authors:** Vandse Aithal, Benjamin O’Sullivan, Ross Prager

**Affiliations:** 1 Renal Department, Nephrologist Morriston Hospital, Swansea, Wales; 2 Acute Medicine Department, FAMUS supervisor, FUSIC mentor, Glangwili Hospital, Carmarthen, Wales; 3 Division of Critical Care, Western University, London, ON Canada

**Keywords:** Venous Excess Ultrasound, Point of Care Ultrasound, Left Ventricular Outflow Velocity Time Integer, Echocardiography

## Abstract

**Background:**

Fluid removal during hemodialysis plays a vital role in achieving optimal patient outcomes. Traditional methods to guide dialysis prescription by estimating dry weight are often sufficient, however, lack precision when assessing the nuanced interplay of venous and arterial physiology in hemodynamically complex patients. Left ventricular outflow tract velocity–time integral (LVOT VTI) provides a dynamic marker of forward stroke volume and can offer additional insight into intravascular volume status when conventional ultrasound markers of congestion are limited. LVOT VTI reflects effective left ventricular forward flow and is sensitive to changes in preload. LVOT VTI may improve with judicious fluid removal despite intradialytic hypotension, offering a practical physiological target to guide ultrafiltration in patients with challenging hemodynamics.

**Case presentation:**

We present a 70-year-old gentleman with end-stage renal disease (ESRD) who switched to hemodialysis in September 2021 from peritoneal dialysis (PD) due to poor ultrafiltration, resulting in persistent fluid overload, including recurrent pleural effusions. An echocardiogram performed in October 2019 revealed significant pulmonary hypertension, severe tricuspid regurgitation (TR), and moderate mitral regurgitation (MR). Fluid management during hemodialysis proved challenging due to persistent predialysis hypotension and further drops in blood pressure during dialysis sessions. To address these challenges and guide fluid removal more precisely, we utilised point-of-care ultrasound (POCUS) to monitor the patient’s volume status. By prioritising stroke volume surrogates, specifically velocity-time integrals (LVOT VTI), over blood pressure as a guide for fluid removal, we were able to safely increase fluid removal per session and reduce his dry weight by 4 kg over 4 weeks. This was accompanied by significant improvement in his symptoms from fluid overload.

**Conclusion:**

Fluid removal was guided by POCUS to address the patient’s complex hemodynamics. Despite intradialytic hypotension, we observed a significant increase in the patient’s LVOT VTI with ongoing fluid removal. This metric may serve as an adjunctive tool to guide dialysis prescription for select cases.

## Background

Fluid removal on hemodialysis is crucial to patient outcomes for patients with end-stage renal disease. Inadequate fluid removal is associated with refractory hypertension (hypertension despite being on 3 antihypertensives) with consequent left ventricular hypertrophy, chronic pulmonary congestion, pleural effusions and volume dependent pulmonary hypertension.

Traditionally, clinical assessment and measurement of dry weight determines the amount of interdialytic fluid removed. Clinical assessment of dry weight relies heavily on signs of congestion with a paucity of clinical markers to assess forward flow. Systemic hypertension is considered an indication of increased intravascular volume and prompts fluid removal on dialysis. Hypotension is thought to reflect a drop in forward flow and limits fluid removal on dialysis, precipitating a spiral of inadequate fluid removal in the face of congestion especially in patients with complex cardiac physiology. This leaves clinicians in need of reproducible metrics that are individualised to guide dialysis prescription in this patient cohort. One of these tools is point of care ultrasound (POCUS).

The utility of POCUS (point-of-care ultrasound) to guide dialysis prescriptions has shown variable results in the literature. One challenge with traditional paradigms of POCUS to guide volume management has been their focus on either forward flow (stroke volume surrogates like velocity time integral (VTI)) or congestion (B-lines or Doppler level assessment using VeXUS) [1]. While individually useful in certain cases, these paradigms fail to consider the interplay between both arterial (forward flow) and venous (congestive) forces. For patients with complex hemodynamics, both inadequate forward flow and congestion can lead to adverse outcomes.

The interpretation of the VEXUS (Venous Excess Ultrasound) score in haemodialysis patients with severe tricuspid regurgitation is difficult given its dependence on congestion alone. There is limited data on its use in patients on haemodialysis with only a small percentage of the study patients registering a high congestion score on VEXUS [2]. 

Similar approaches have utilized lung ultrasound to evaluate B-lines, which indicate pulmonary congestion and indirectly reflect left atrial pressures [3]. Additionally, Doppler assessments of the portal vein have been explored in patients with tricuspid regurgitation, but findings reveal limited improvement rates in patients with severe Tricuspid Regurgitation, with complete resolution observed in 41% of cases and partial improvement in 38% [4].

Other POCUS-based approaches utilising measurement of inferior vena cava (IVC) size to assess volume status [5] are unreliable in patients with right sided heart disease and tricuspid regurgitation. Carotid Doppler–based indices have been explored as an alternative means of assessing arterial flow and haemodynamic change [6], but these measurements are also influenced by cardiovascular and vascular pathology commonly seen in patients on dialysis.

LVOT VTI is commonly used as a surrogate for stroke volume because it reflects the velocity-time integral of blood flow through the left ventricular outflow tract, directly correlating with the volume of blood ejected with each heartbeat.

By integrating LVOT VTI into dialysis prescriptions, clinicians can shift the focus toward optimizing forward flow and left ventricular performance rather than solely addressing right-sided congestion, potentially leading to more personalized and effective treatments.

In this case report, we describe a hemodynamically complex patient on hemodialysis with severe pulmonary hypertension, tricuspid regurgitation, and mitral regurgitation. We highlight the value of a comprehensive hemodynamic profile by utilizing the left ventricular outflow tract (LVOT) velocity-time integral (VTI) to non-invasively assess stroke volume, while considering right sided congestion through examining the portal vein.

## Case presentation

A 70 year old gentleman who developed ESRD on a background of Congenital anomaly of the kidney and urinary tract (CAKUT) became dialysis dependent in 2019. There was a history of bilateral hydronephrosis noted in the neonatal period. When he presented to us in 2014, he had small kidneys with multiple scars in both kidneys. He had long-standing hypertension. There was no history of diabetes, vascular disease or amyloidosis. He was not a smoker and his BMI was 26. 

He initially started peritoneal dialysis in March 2019. He had evidence of LVH on the echocardiogram in September 2018. He had poor ultrafiltration on PD and developed persistent fluid overload including persistent pleural effusions and the Echocardiogram in Oct 2019 showed significant pulmonary hypertension, severe TR and moderate MR. He was hypotensive at this stage and was switched to hemodialysis in Sept 2021. He was switched to haemodialysis primarily because he had become oligoanuric and peritoneal dialysis was not able to facilitate adequate fluid removal.

Fluid removal on hemodialysis was complicated by persistent hypotension pre dialysis with further drops during dialysis. The blood pressure pre dialysis was consistently around 90/60 with intradialytic drops to 60/40 mmHg. The post dialysis blood pressures did always improve to the pre dialysis blood pressure range. The systemic hypotension was secondary to significant pulmonary hypertension, severe tricuspid regurgitation and moderate to severe mitral regurgitation with consequent reduction in left ventricular stroke volume.

He benefited from an extra fourth session per week to remove fluid and there was an improvement in his functional reserve and in his overall fluid balance including the size of the pleural effusions. Fluid assessment however was complicated by the presence of severe TR and pulmonary hypertension that persisted despite significant drops in his dry weight. His dry weight had been gradually reduced from 73 to 60 kg over a 12-month period and further reduction was not possible as the patient became increasingly symptomatic from intra-dialytic hypotension. Pre-dialysis pedal oedema was no longer present but he had a persistently elevated jugular venous pressure and evidence of a right pleural effusion. There were therefore no clinical variables that could be used to guide further reduction in his dry weight.

We used POCUS to assess fluid status before and after dialysis sessions. A phased array probe was used to determine pre-dialysis and post-dialysis LVOT VTI as a surrogate for stroke volume. POCUS demonstrated an increase in LVOT VTI with ongoing volume removal, implying that stroke volume improved despite drops in blood pressure (Table 1). In addition there was a reduction in right ventricular (RV) size and mitral valve regurgitation, along with an improved E/A ratio after each dialysis session (Table 2). This allowed us to increase ultrafiltration during dialysis despite intradialytic systolic hypotension due to the fact that the LVOT VTI was improving. We were able to reduce his target by 4 kg over 4 weeks. The patient reported improvement in quality of life at this time. Of note, some echocardiographic features did not change: severity of tricuspid regurgitation, portal vein pulsatility, and VeXUS score.

The patient reported an improvement in the following symptoms: Shortness of breath, sleep, appetite, energy. He did not report any cramps or dizziness. Based on these findings, his condition was reclassified from New York Heart Association (NYHA) Class II to Class I heart failure.

**Table 1:** Study data. (Blood pressure taken directly before and after dialysis)

	16/08-pre dialysis	16/08-post dialysis	30/08-pre dialysis	30/08-post dialysis	02/09-pre dialysis	02/09-post dialysis	06/09-pre dialysis	06/09-post dialysis	11/10-Pre dialysis	11/10-Post dialysis
Left Ventricle Outflow Tract Velocity Time Integral (LVOT VTI)	14.7	21.5	20.3	21	19.29	20.32	18.53	21.74	15.39	18.92
Cardiac Output (CO)	3.56	4.02	3.44	3.49	3.4	4.29	4.36	5.11	2.56	4.45
Stroke Volume (SV)	50.86	74.39	70.24	72.66	66.74	70.31	64.11	75.22	53.25	65.46
Heart Rate (HR)	70	54	49	48	51	61	68	68	48	68
Volume lost		2.4 L		1.3 L		1.5 L		1.4 L		1.6 L
Weight	61.2 kg	59.7 kg	57.4 kg	56.1 kg	58.4 kg	56.9 kg	57.5 kg	56.1 kg	57.8 kg	56.2 kg
Blood Pressure (BP)	88/45	72/48	84/50	66/45	93/50	71/45	81/48	67/42	97/53	83/41
Portal Vein pulsatility (VeXUS)	>50%	>50%	> 50%	50%	> 50%	> 50%	> 50%	> 50%	> 50%	> 50%
Inferior Vena Cava (VeXUS)	Plethoric IVC > 2.5, non collapsible	Plethoric IVC > 2.5, non collapsible	Plethoric IVC > 2.5, non collapsible	Plethoric IVC > 2.5, non collapsible	Plethoric IVC > 2.5, non collapsible	Plethoric IVC > 2.5, non collapsible	Plethoric IVC > 2.5, non collapsible	Plethoric IVC > 2.5, non collapsible	Plethoric IVC > 2.5, non collapsible	Plethoric IVC > 2.5, non collapsible
Hepatic Vein (VeXUS)	S wave reversal	S wave reversal	S wave reversal	S wave reversal	S wave reversal	S wave reversal	S wave reversal	S wave reversal	S wave reversal	S wave reversal

**Table 2:** E/A, RV/LV ratio, MR

	06/09-pre dialysis	06/09-post dialysis	11/10-Pre dialysis	11/10-Post dialysis
Right VentricleV/Left Ventricle ratio	1.27	1.25	1.17	1.16
E/A (E= Early ventricular filling, A= Atrial contraction).	1.37	1.13	3.56	3.06
Mitral Regurgitation (MR)	Mod	Mild	Mod	Mild

### Measurements not affected by dialysis


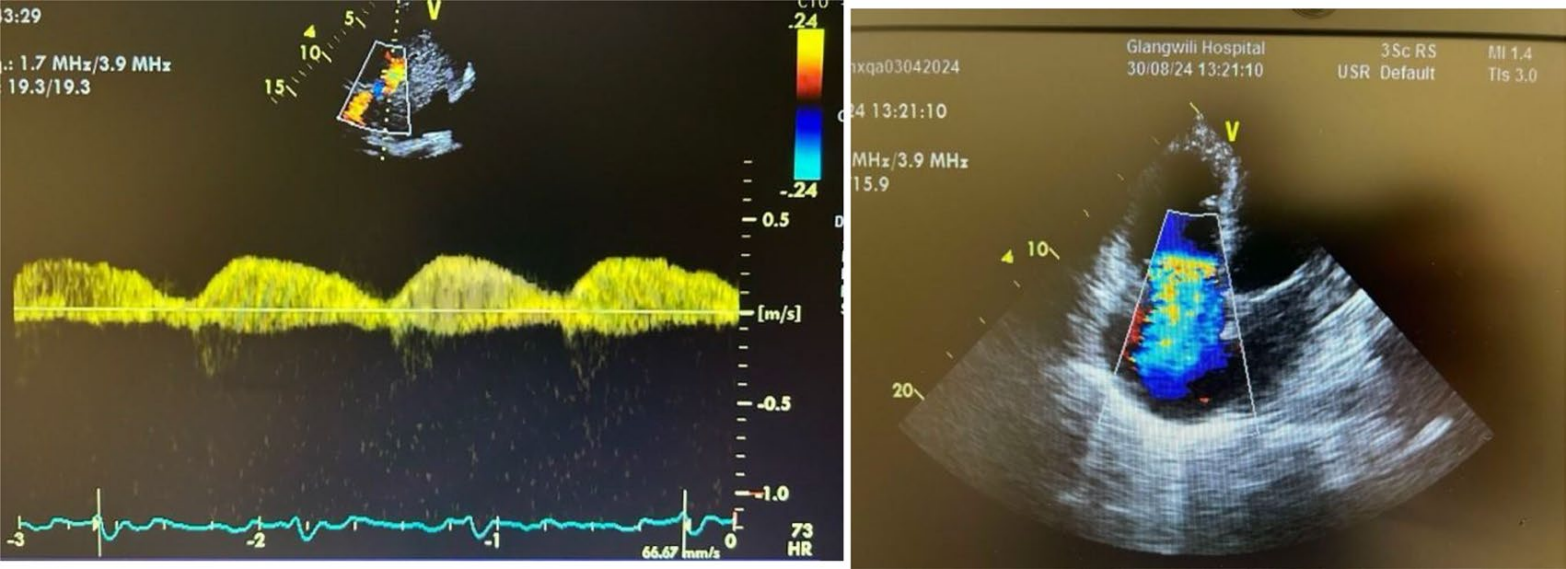


Image 1: Pulsatile portal vein > 50% (left). Image 2: Severe torrential Tricuspid Regurgitation with dilated Right ventricle (right).

### Factors that show change before and after dialysis


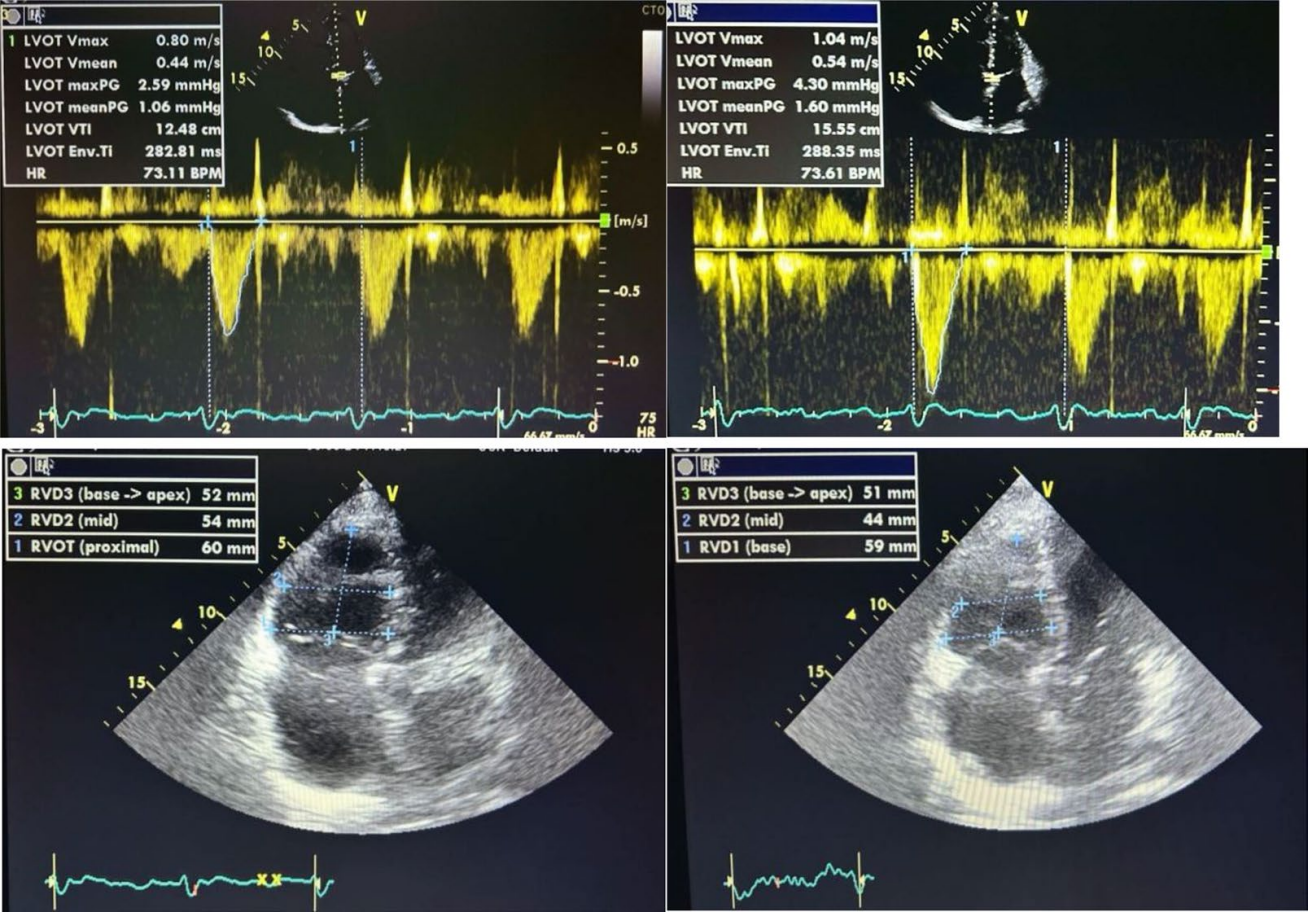


Top: Image 3: Pre dialysis LVOT VTI (left). Image 4: Post dialysis LVOT VTI (right). Bottom: Image 5: RV dimensions before (left) and Image 6: after dialysis (right).

## Discussion

This case emphasizes the need for individualised intradialytic resuscitation and fluid removal strategies guided by echocardiographic metrics. A key part of this approach is to find reproducible measures of forward flow that would allow fluid removal despite worsening hypotension in a patient with heart failure on haemodialysis.

Bou Chebl et al. showed in an observational study that LVOT VTI in conjunction with passive leg raise correlated with volume status and volume responsiveness in patients on haemodialysis [7].

LVOT VTI, a surrogate for forward flow, proved to be a more practical and informative guide than blood pressure alone in our patient.

Although portal vein pulsatility in conjunction with LVOT VTI offers a more comprehensive view of the patient’s volume status, it remained consistently above 50% in our case, likely reflecting severe tricuspid regurgitation (TR). This finding aligns with studies, such as those by Argaiz et al., which demonstrate that portal vein pulsatility rarely improves beyond 50% in cases of severe TR.

Right ventricular volume overload is known to reduce left ventricular diastolic function, even in the presence of preserved contractility. Over time, right ventricular pressure overload compromises left ventricular compliance and may cause atrophic remodeling, ultimately leading to reduced systolic function [8]. In our patient, both right ventricular volume and pressure overload were evident on echocardiography. The improvement in LVOT VTI likely reflects a reduction in ventricular interdependence facilitated by decreased right ventricular pressure through fluid removal. (Table 2)

Additionally, we noted a reduction in mitral regurgitation, which may have improved left ventricular preload by decreasing regurgitant volume and enhancing forward flow efficiency. Given these findings, we considered nocturnal hemodialysis with slower ultrafiltration rates, a strategy known to improve cardiovascular outcomes [9]. However, the patient declined this option due to personal preferences, underscoring the practical challenges of implementing new dialysis strategies in routine care.

By integrating forward flow measurements with assessments of right-sided congestion, this case illustrates a potential for phenotyped dialysis prescriptions that go beyond traditional reliance on blood pressure and dry weight.

## Conclusion

This case highlights a scenario of complex hemodynamics where the portal vein was unable to provide actionable insights into decongestion, necessitating a shift in focus toward evaluating forward flow.

## Patient perspective

In discussion with the renal team I had more fluid removed during haemodialysis with the help of the scans before and after dialysis. I didn’t experience any untoward symptoms during my dialysis sessions despite low blood pressures that I normally experience during dialysis. I noticed that after a couple of sessions of taking off more fluid that my sleep improved due to not feeling short of breath and I also had more energy to do activities such as working out and I am now able to lift light weights at home.

## Data Availability

Raw data and images attached to documents, but real time images are available on echo machine. Email benosullivan92@Outlook.com for access to images.

## Abbreviations

POCUS: Point of Care Ultrasound
VTI: Velocity Time Integral
